# New Quinoxaline Derivatives as Potential MT_1_ and MT_2_ Receptor Ligands

**DOI:** 10.3390/molecules17077737

**Published:** 2012-06-25

**Authors:** Saioa Ancizu, Nerea Castrillo, Silvia Pérez-Silanes, Ignacio Aldana, Antonio Monge, Philippe Delagrange, Daniel-Henry Caignard, Silvia Galiano

**Affiliations:** 1 Unidad en Investigación y Desarrollo de Medicamentos, Centro de Investigación en Farmacobiología Aplicada (CIFA), Universidad de Navarra, C/Irunlarrea, 1, 31008 Pamplona, Spain; Email: sancizupere@alumni.unav.es (S.A.); ncastrillo@alumni.unav.es (N.C.); sperez@unav.es (S.P.-S.); ialdana@unav.es (I.A.); amonge@unav.es (A.M.); 2 IdRServier, 125 Chemin de ronde, 78290 Croissy-sur-Seine, France; Email: philippe.delagrange@fr.netgrs.com (P.D.); daniel-henri.caignard@fr.netgrs.com (D.-H.C.)

**Keywords:** sleep disorders, melatonin, MT_1_/MT_2_ receptors, quinoxalinamide, quinoxalinurea

## Abstract

Ever since the idea arose that melatonin might promote sleep and resynchronize circadian rhythms, many research groups have centered their efforts on obtaining new melatonin receptor ligands whose pharmacophores include an aliphatic chain of variable length united to an *N-*alkylamide and a methoxy group (or a bioisostere), linked to a central ring. Substitution of the indole ring found in melatonin with a naphthalene or quinoline ring leads to compounds of similar affinity. The next step in this structural approximation is to introduce a quinoxaline ring (a bioisostere of the quinoline and naphthalene rings) as the central nucleus of future melatoninergic ligands.

## 1. Introduction

The frequent pathology of sleep disorders has diverse origins. The clinical manifestations vary and may include insomnia, hypersomnia, respiratory disorders, complex motor disorders, *etc*. These disorders can appear alone or associated to each other, thereby making this a very complex pathology. Insomnia is the most frequent disorder within the general population [1]. The complaints from more than 50% of primary care patients are related to insomnia and an estimated 9 to 18% of the adult population suffers from chronic insomnia [2,3].

In the regulation of the awake-sleep system, a complex neural network is involved, in which different areas of the brain are activated and inhibited, following a circadian rhythm that lasts 24 h. The suprachiasmatic nucleus (SCN) is responsible for synchronizing the circadian rhythm as well as for promoting sleep, by means of melatonin (MLT), the main hormone secreted by the pineal gland [4,5]. In fact, it has been observed that administration of MLT results in an increase of sleepiness. 

Melatonin regulates sleep and the circadian rhythms by means of its receptors MT_1_ and MT_2_ which in recent years have become one of the most interesting pharmacological targets [4,6].The diversity and distribution of MT_1_ and MT_2_ in the different tissues indicate that each one of these receptors possibly possesses a different physiological function, although this possibility must be more thoroughly studied. Some studies suggest that the effect of promoting sleep is related to the suppression of activity in the SCN neurons by means of activation of the receptors MT_1_. Other studies report that the phase changes of the neuronal rhythm are related to the activation of the receptors MT_2_ at dawn [4,7]. 

**Figure 1:**
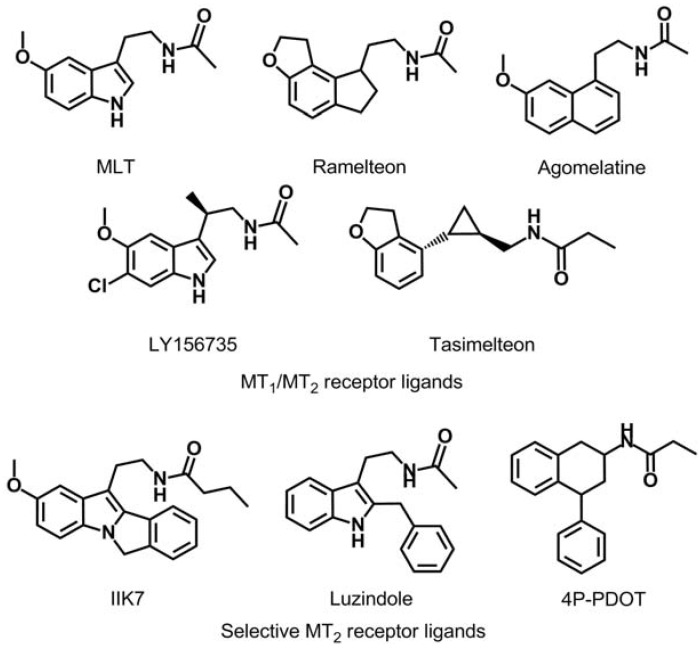
Chemical structures of MT_1_/MT_2_ receptors agonist and MT_2_-selective ligands.

Ever since the idea arose that MLT might promote sleep and resynchronize circadian rhythms, many research groups have centered their efforts on obtaining new melatonin receptor ligands [4,6,8,9,10,11]. As can be observed in Figure 1, several series of MT_2_ ligands have been described as a competitive agonists or antagonists with varying degrees of selectivity (IIK7 is a selective MT_2_ receptor agonist and luzindole and 4P-PDOT are a selective MT_2_ receptor antagonists) as well as compounds with affinity for both melatoninergic receptor subtypes (melatonin, ramelteon, agomelatine, LY156735 and tasimelteon) [12,13,14,15]. 

Recently MT_2_ receptor subtype selectivity has registered some important advances that could result in a safer compound with a more favorable pharmacological profile [16,17,18,19]. During the last two decades, numerous research groups have sought to understand how melatonin interacts with its receptors. A number of structure-activity relationships (SAR) have been studied proposing molecular models of the melatonin binding site and 3D pharmacophore models [20,21,22,23,24,25,26,27]. These models have been reviewed and completed in recent reviews [6,28].

A brief description of the general SAR concepts developed in these decades follows. The pharmacophore structure that can be found in almost all MLT receptor agonists is composed by an amide group connected by a linker chain of variable length to an aromatic nucleus (indole or a bioisostere) carrying a methoxy group, or a bioisostere, such as bromine.

**Figure 2:**
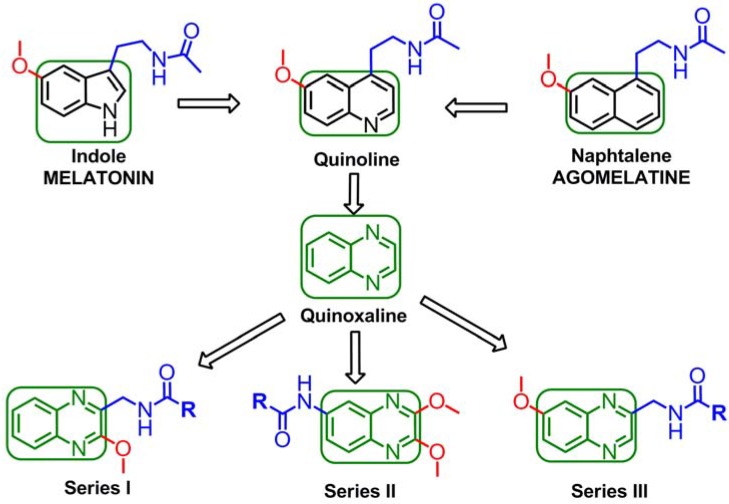
Structural approach of the indole ring and the naphthalene ring to quinoline ring and to quinoxaline ring.

Since the indole ring of MLT is not essential for binding to the receptor, several potent ligands have been designed by employing bioisosteric replacement of the indole moiety by other aromatic rings. Substitution of the indole ring with the naphthalene ring system leads to agomelatine, which presents higher affinity for MT_1_/MT_2_ than MLT. In addition, replacement of the naphthalene ring with a quinoline scaffold leads to compounds of similar affinity [29,30]. The bioequivalency disclosed between the indole moiety replaced by naphthalene and quinolone rings is a clear example of a structural approach, in which the next logical step was to introduce the quinoxaline ring as shown in Figure 2. The quinoxaline derivatives represent a privileged chemical structure because they possess important biological properties with applications in diverse therapeutic fields as anticancerous [31], anti-inflammatory/antioxidant [32], antibacterial [33] and sedative and anticonvulsant agents [34]. Thus, in this study, we decided to design and synthesize quinoxaline derivatives in order to verify whether the quinoxaline is a valuable heterocyclic bioisostere of the melatonin indole nucleus.

In addition, early SAR studies showed that both the 5-methoxy group and the *N*-acetylamino side chain of melatonin are crucial for high receptor affinity and that the relative spatial distance between these groups is also an important factor [35]. However, based on extended SAR data of a series of tryptamines, it has been concluded that the 5-methoxy group is not an essential requirement for biological activity, although it clearly shows major interactions with a specific binding site in the melatonin receptor for a broad variety of ligands [36] and it is known to increase potency by forming a hydrogen bond with His211 in the putative transmembrane domain 5 (TM5) [37,38].

Moreover, it has been reported that appropriate substituents *ortho* to the ethylamido side chain in the naphthalenic melatonin analogues modulate the binding affinity to the melatonin receptor [39]. Thus, we were further interested in the effects of switching the methoxy group from position 6 of the quinoxaline scaffold (analogous to position 5 in the melatonin indole ring) to position 2, contiguous to the *N*-acylamido side chain. Herein, we report a preliminary design, synthesis and biological evaluation of three novel series of quinoxaline derivatives as shown in Figure 2.

## 2. Results and Discussion

### 2.1. Chemistry

**Scheme 1:**
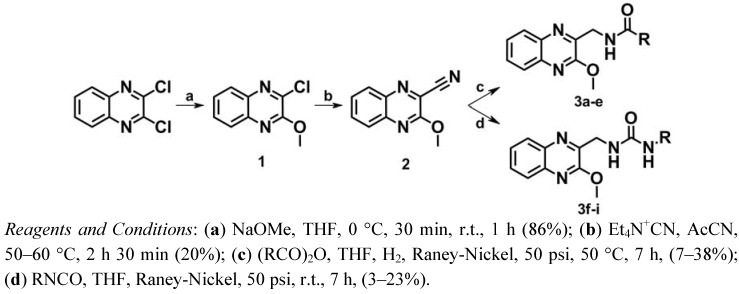
Synthesis of series I.

The synthetic route followed for the synthesis of series I is shown in Scheme 1. First, one of the chlorine atoms of 2,3-dichloroquinoxaline was replaced by a methoxy group using sodium methoxide in tetrahydrofuran (THF) leading to compound **1** [40]. According to the procedure described by Herrman *et al.* [41,42] a nitrile group was then introduced into position 2 of the quinoxaline by treatment with tetraethylammonium cyanide in the presence of acetonitrile at 50–60 °C to give compound **2**. 

The amide derivatives **3a**–**e** were obtained according to previously described methods [43,44]. Hydrogenation of the nitrile group of **2** over Raney-Nickel in THF and concomitant *N-*acylation with a suitable anhydride gave the desired amide derivatives. In the case of the urea derivatives **3f**–**i**, several trials and strategies were carried out. At the beginning, a classical two-step strategy was designed in which a catalytic hydrogenation of the nitrile group of **2** over Raney-Nickel in ammonia and ethanol provided the desired amines which were converted to urea by reaction of the primary amine with the corresponding isocyanate [45]. Due to the failure of this strategy, further investigations regarding these reactions led to the development of a novel one-step procedure. Reduction of the nitrile group of **2** by hydrogen over Raney-Nickel in THF and concomitant addition of an appropriate isocyanate gave the desired urea derivatives mixed with the hydrolyzed isocyanates, as expected.

**Scheme 2:**
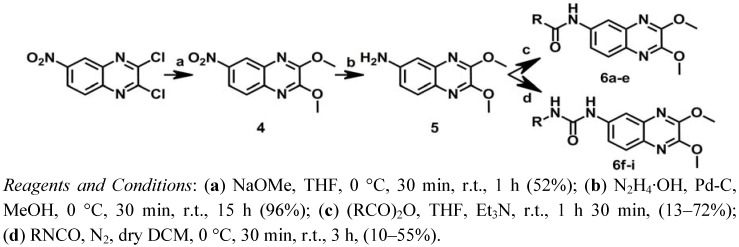
Synthesis of series II.

With the aim of obtaining the compounds of series II, a new synthetic route was designed, as shown in Scheme 2. 2,3-Dichloro-6-nitroquinoxaline was first treated with sodium methoxide in THF to give **4** [16]. Subsequently, compound **5** was obtained through the reduction of the nitrile group of **4**with hydrazinium hydroxide and palladium on carbon [46]. Finally, *N-*acylation of compound **5** using a suitable anhydride and triethylamine in THF yielded the corresponding amide analogs **6a**–**e** [47], while treatment of **5** with different isocyanates in dichloromethane (DCM) afforded the urea analogs **6f**–**i** [45].

**Scheme 3:**
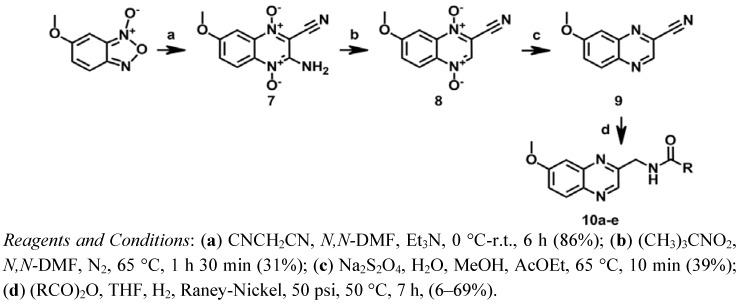
Synthesis of series III.

Compounds **10a**–**e** of series III were synthesized as depicted in Scheme 3. A modified Beirut reaction was adopted for the preparation of **7** by condensation of 6-methoxybenzofuroxane and malononitrile. 

Next, compound **7** was deaminated leading to compound **8** by means of the corresponding diazonium salt intermediate, using *tert*-butyl nitrite in *N,N-*dimethylformamide (*N,N-*DMF) as previously described [48]. Treatment of compound **8** with sodium dithionite in water in the presence of methanol (MeOH) and ethyl acetate (AcOEt) provided compound **9** [32,49]. Finally the amide derivatives, **10a**–**e**, were obtained by the hydrogenation of the nitrile group of **9** over Raney-Nickel in THF and concomitant *N-*acylation with a suitable anhydride as previously described [43,44].

### 2.2. Pharmacology and Structure-Activity Relationship

The binding affinity of the newly synthesized quinoxalines for MT_1_ and MT_2_ receptors was tested through competitive binding experiments using 2-[^125^I]Iodomelatonin as radioligand. Biological evaluation was performed according to previously described methods [50] and the obtained results are reported in Table 1. 

**Table 1:** MT_1_ and MT_2_ binding affinities (µM) of new synthesized Quinoxalines.

Quinoxalines	Comp.	R	MT_1_ Ki (µM) ± SEM	MT_2_ Ki (µM) ± SEM
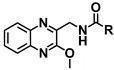	**3a**	CH_3_	2.98 ± 0.36	0.88 ± 0.30
	**3b**	CH_2_CH_3_	2.60 ± 0.23	0.66 ± 0.01
	**3c**	CH_2_CH_2_CH_3_	0.75 ± 0.36	1.10 ± 0.03
	**3d**	CH(CH_3_)_2_	1.23 ± 0.32	0.47 ± 0.02
	**3e**	Ph	>10^3^	>10^3^
	**3f**	NHCH_2_CH_3_	>10^3^	0.40 ± 0.13
	**3g**	NHCH_2_CH_2_CH_3_	>10^3^	0.44 ± N.D.
	**3h**	NHCH(CH_3_)_2_	>10^3^	>10^3^
	**3i**	NHPh	>10^3^	>10^3^
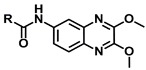	**6a**	CH_3_	20.00 ± 1.82	0.08 ± N.D.
	**6b**	CH_2_CH_3_	17.60 ± 7.81	4.36 ± 1.22
	**6c**	CH_2_CH_2_CH_3_	11.50 ± 2.80	1.35 ± 0.31
	**6d**	CH(CH_3_)_2_	3.40 ± 1.37	10.50 ± 2.96
	**6e**	Ph	>10^3^	>10^3^
	**6f**	NHCH_2_CH_3_	3.41 ± 1.89	28.80 ± N.D
	**6g**	NHCH_2_CH_2_CH_3_	1.63 ± 0.44	0.489 ± 0.07
	**6h**	NHCH(CH_3_)_2_	>10^3^	>10^3^
	**6i**	NHPh	>10^3^	>10^3^
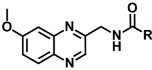	**10a**	CH_3_	>10^3^	>10^3^
	**10b**	CH_2_CH_3_	>10^3^	0.34 ± 0.15
	**10c**	CH_2_CH_2_CH_3_	0.21 ± 0.11	0.10 ± 0.01
	**10d**	CH(CH_3_)_2_	0.32 ± 0.04	0.16 ± 0.00
	**10e**	Ph	>10^3^	>10^3^
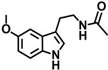	**MLT**		0.14·× 10^−3^ ± 0.03·× 10^−3^	0.41·× 10^−3^ ± 0.04·× 10^−3^

Binding affinities (μM) are expressed as mean Ki ± SEM of at least three independent experiments. N.D. not determined.

As can be observed in Table 1, compounds **10c** and **10d** of Series III present the best affinity profile towards MT1/MT2 receptors. Comparing these data with their analogues **3c** and **3d** of Series I, it can be concluded that the existence of the six-atom distance between the methoxy group and the first nitrogen atom of the side chain is essential for obtaining a good melatoninergic MT_1_/MT_2_ binding affinity. Derivative **10c** exhibited the best melatoninergic binding affinity with both receptors and therefore, this compound could be selected as a hit in the search for new and more active quinoxalines acting as MT_1_/MT_2_ receptor ligands. 

As shown by compounds of Series I and II, shifting the methoxy group from position C6 to C2 of the quinoxaline moiety can modulate the affinity and selectivity of these ligands depending on the distance between the methoxy group and the *N*-acylamino chain. The comparison of the obtained data for compounds of both series and Series III reveals that switching the methoxy group to position 2 of the quinoxaline ring has a much greater effect on binding affinity to the MT_2_ receptor subtype than it does on affinity to MT_1_ receptor, displaying a significant selectivity for MT_2_ receptor in Series II, where the relative spatial distance (six-atoms) between the two key pharmacophoric elements is also the optimal.

Compound **6a** showed the most promising profile in terms of affinity and selectivity for the MT_2_ receptor with MT_2_/MT_1_ selectivity ratio of 250 and the highest binding affinity (0.08 μM) for the MT_2_ receptor. These results indicate that compound **6a** is a potent and selective MT_2_ ligand. Furthermore, the functional activity of two of the best compounds of this series (**6c** and **6a**) has been evaluated only on the MT_2_ receptor subtype, due to the weak affinity for the MT_1_ subtype. The results are shown in Table 2. Compound **6c** showed full agonist profile with a EC_50_ = 1.3 μM and E_max_ = 84%. In the case of compound **6a**, due to the fact that this compounds exhibited an EC_50_ > 10 μM, the compound **6a** might considered as an antagonist.

**Table 2:** MT_2_ GTPγS (+) binding affinities of compounds **6a** and **6c**.

Compound	MT_2_	
	EC_50_ ± SEM (μM)	E_max_ ± SEM (%)
MLT	0.49 ×·10^−3^ ± 0.05·× 10^−3^	100
**6a**	>10	
**6c**	1.3 ± 0.26	84 ± 9.5

Concentration-response curves were analysed by non-linear regression. Agonist potency was expressed as EC_50_ ± SEM (μM) while the maximal efficacy, E_max_ ± SEM was expressed as a Percentage of that observed with melatonin 1 μM (100%).

With regard to the amide/urea substitution, the results are inconclusive, therefore, it cannot be determined which group (amide/urea) leads to better affinity for MT_1_/MT_2_ receptors. Comparing the results obtained from Series I, it could be assumed that the amide derivatives **3a**–**e** generally present better affinity for MT_1_ receptor than their homologous urea derivatives **3f**–**i**. However, the affinity of urea derivatives for MT_2_ is higher than the affinity of amide derivatives for this same receptor, provided that the aliphatic chain linked to the urea/amide group is a linear aliphatic chain. In contrast, taking into account the results obtained for the compounds from Series II, it could be concluded that the urea derivatives **6a**–**e** present higher affinity for both receptors than their homologous amide derivatives **6f**–**i**, provided that the aliphatic chain linked to the urea/amide group is a linear aliphatic chain. Thus, it can be said that the introduction of a urea function, instead of the amide function existing in the MLT, does not negatively affect the affinity of the ligands for MT_1_ and MT_2_ receptors. In addition, since it is already well known that urea derivatives usually present better metabolic stability than amide derivatives, the urea derivatives are preferred. 

Finally, the introduction of a benzene ring, replacing the aliphatic chain substituted over the amide/urea group, results in a total loss of affinity. By contrast, substitution with an aliphatic linear chain in general, and particularly with a propyl group, appeared to be the best option in order to obtain high affinity compounds.

**Figure 3:**
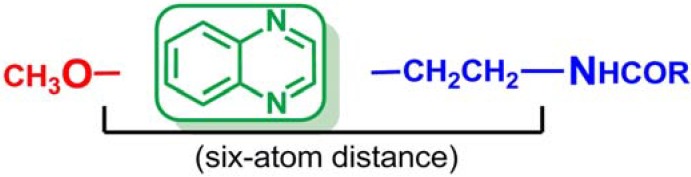
Structural requirements of future series of quinoxalines as MT_1_/MT_2_ receptor ligands.

With these results, we were not able to state that the quinoxaline scaffold can act as a useful bioisostere of indole in the search for melatonin receptor ligands. In addition, The SAR study suggested that a six-atom length is the best distance between the methoxy group and the first nitrogen atom of the side chain in order to obtain high MT_1_/MT_2_ affinity. Moreover, new bibliographic review has revealed that the optimum distance should be obtained through a two-methylene linker in order to allow compounds to adopt a MLT-like configuration [51]. Thus, the structural requirements initially described have been redefined and the synthesis of a new series of quinoxalines has been proposed in which the aforementioned distance is obtained through a two-methylene linker (Figure 3). These conclusions open a new line of research in order to determine if the quinoxaline ring is suitable as central scaffold of melatoninergic ligands.

## 3. Experimental

### 3.1. Chemical Synthesis

#### 3.1.1. General Remarks

All of the synthesized compounds were chemically characterized by thin layer chromatography (TLC), infrared (IR), proton nuclear magnetic resonance (^1^H-NMR) and elemental microanalyses (CHN). Alugram SIL G/UV254 (Layer: 0.2 mm) (Macherey-Nagel GmbH & Co. KG., Düren, Germany) was used for TLC, and Silica gel 60 (0.040–0.063 mm, Merck, Darmstadt, Germany) was used for flash column chromatography. The ^1^H-NMR spectra were recorded on a Bruker 400 Ultrashield instrument (Bruker, Rheinstetten, Germany) 400 MHz), using TMS as internal standard and with DMSO-d_6_ or CDCl_3_ as solvents; the chemical shifts are reported in ppm (*δ*) and coupling constant (*J*) values are given in Hertz (Hz). Signal multiplicities are represented by: s (singlet), bs (broad singlet), d (doublet), dd (doublet of doublets), ddd (doublet of doublet of doublets), t (triplet), dt (doublet of triplets), tt (triplet of triplets); q (quartet); dq (doublet of quartets) and m (multiplet). The IR spectra were recorded on a Nicolet Nexus FTIR (Thermo, Madison, WI, USA) in KBr pellets. Elemental microanalyses were obtained on a CHN 900 Elemental Analyzer (Leco, Tres Cantos, Spain) from vacuum-dried samples. The analytical results for C, H and N, were within ±0.4 of the theoretical values. Chemicals were purchased from Panreac Química S.A. (Montcada i Reixac, Spain), Sigma-Aldrich-Fluka Química S.A. (Alcobendas, Spain), Alfa Aesar-Avocado GmbH & Co. KG (Karlsruhe, Germany), and E. Merck (Darmstadt, Germany).

#### 3.1.2. Synthesis of 2-Chloro-3-methoxyquinoxaline (**1**)

2,3-Dichloroquinoxaline (90%, 2.30 g, 10.04 mmol) and THF (20.00 mL) were placed in a flask. The mixture was cooled to 0 °C. A solution of sodium methoxide was obtained from the reaction between Na (0.50 g) and methanol (5.00 mL). NaOMe (2.34 mL, 10.04 mmol) was added dropwise to the other suspension. The color of the mixture changed from purple to yellow. The mixture was stirred during 30 min at 0 °C and during 1 h at room temperature. DCM (80.00 mL) was added and the mixture was quenched with brine. The DCM solution was dried over Na_2_SO_4_ and filtered. The solvent was removed under reduced pressure to give compound **1** as a yellow solid. Yield: 86%. IR (cm^−1^): 3032, 2987, 2936. ^1^H-NMR (CDCl_3_) *δ*: 7.96 (ddd, 1H, **H_8_**, *J_8-7_* = 8.3 Hz, *J_8-6_* = 1.5 Hz, *J_8-5_ =* 0.5 Hz); 7.88 (ddd, 1H, **H_5_**, *J_5-6_* = 8.3 Hz, *J_5-7_ =* 1.4 Hz, *J_5-8_ =* 0.5 Hz); 7.71 (ddd, 1H, **H_6_**, *J_6-5_ =* 8.4 Hz, *J_6-7_ =* 7.1 Hz, *J_6-8_ =* 1.5 Hz); 7.60 (ddd, 1H, **H_7_**, *J_7-8_ =* 8.4 Hz, *J_7-6_ =* 7.1 Hz, *J_7-5_ =* 1.5 Hz); 4.20 (s, 3H, OC**H_3_**) ppm. Anal. Calcd for C_9_H_7_N_2_OCl: C: 55.53%; H: 3.60%; N: 14.40%; Found: C: 55.23%; H: 3.34%; N: 14.41%. 

#### 3.1.3. Synthesis of 3-Methoxyquinoxaline-2-carbonitrile (**2**)

Compound **1** (1.59 g, 8.17 mmol) and tetraethylammonium cyanide (2.55 g, 16.32 mmol) were dissolved in acetonitrile (60.00 mL). The mixture was heated between 50–60 °C during 2 h 30 min. The solution was poured onto ice and a black precipitate was formed which was purified by silica gel column chromatography using DCM as mobile phase. The solvent was removed under reduced pressure to yield compound **2**. Yield: 20%. IR (cm^−1^): 3026, 2943, 2232. ^1^H-NMR (CDCl_3_) *δ*: 8.08 (ddd, 1H, **H_8_**, *J_8-7_* = 8.4 Hz, *J_8-6_* = 1.5 Hz, *J_8-5_ =* 0.5 Hz); 7.88 (ddd, 1H, **H_5_**, *J_5-6_* = 8.3 Hz, *J_5-7_ =* 1.4 Hz, *J_5-8_ =* 0.5 Hz); 7.71 (ddd, 1H, **H_6_**, *J_6-5_ =* 8.4 Hz, *J_6-7_ =* 6.9 Hz, *J_6-8_ =* 1.4 Hz); 7.60 (ddd, 1H, **H_7_**, *J_7-8_ =* 8.4 Hz, *J_7-6_ =* 6.9 Hz, *J_7-5_ =* 1.5 Hz); 4.20 (s, 3H, OC**H_3_**) ppm. Anal. Calcd for C_10_H_7_N_3_O: C: 64.86%; H: 3.78%; N: 22.70%; Found: C: 65.23%; H: 3.61%; N: 22.70%.

#### 3.1.4. General Procedure for Synthesis of *N*-(3-Methoxyquinoxalin-2-ylmethyl)-alkylamidea **3a–e**

The previously obtained compound **2**, THF and the corresponding anhydride in proportion 1:2 were placed in a hydrogenation flask. A spatula of Raney-Nickel was added and the mixture was hydrogenated during 7 h at 50 °C and 50 psi. Next, DCM was added and the mixture was decanted off. Next, the mixture was quenched with water, dried over Na_2_SO_4_ and filtered. The solvent was removed under reduced pressure to obtain brown oil. Finally, *n*-hexane was added to the oil and a white solid precipitated, which was filtered and washed with diethyl ether and water. If necessary, it was purified by silica gel column chromatography using DCM/MeOH as mobile phase. The solvent was removed under reduced pressure and the desired amides **3a**–**e** were thus obtained.

*N-(3-Methoxyquinoxalin-2-ylmethyl)-acetamide* (**3a**). Yield: 7%. IR (cm^−1^): 3314, 3084, 2937, 1648. ^1^H-NMR (CDCl_3_) *δ*: 8.11 (dd, 1H, **H_8_**, *J_8-7_* = 8.1 Hz, *J_8-6_* = 1.1 Hz); 7.91 (ddd, 1H, **H_5_**, *J_5-6_* = 8.3 Hz, *J_5-7_ =* 1.3 Hz); 7.71 (ddd, 1H, **H_6_**, *J_6-5_ =* 8.3 Hz, *J_6-7_ =* 7.1 Hz, *J_6-8_ =* 1.3 Hz); 7.62 (ddd, 1H, **H_7_**, *J_7-8_ =* 8.3 Hz, *J_7-6_ =* 7.1 Hz, *J_7-5_ =* 1.4 Hz); 4.76 (d, 2H, C**H_2_**, *J_CH2-NH_* = 4.3 Hz); 4.17 (s, 3H, OC**H_3_**); 2.21 (s, 3H, C**H_3_**) ppm. Anal. Calcd for C_12_H_13_N_3_O_2_ · ^1^/_4_ H_2_O: C: 61.14%; H: 5.73%; N: 17.83%; Found: C: 61.26%; H: 5.47%; N: 17.65%. M.p.: 231–235 °C.

*N-(3-Methoxyquinoxalin-2-ylmethyl)-propionamide* (**3b**). Yield: 27%. IR (cm^−1^): 3308, 3078, 2969, 2943, 1638.^1^H-NMR (DMSO-d_6_) *δ*: 8.27 (t, 1H, N**H**, *J_NH-CH2_* = 5.3 Hz); 7.96 (dd, 1H, **H_8_**, *J_8-7_* = 8.2 Hz, *J_8-6_* = 1.2 Hz); 7.85 (dd, 1H, **H_5_**, *J_5-6_* = 8.3 Hz, *J_5-7_* = 1.0 Hz); 7.72 (ddd, 1H, **H_6_**, *J_6-5_* = 8.3 Hz, *J_6-7_* = 7.0 Hz, *J_6-8_* = 1.5 Hz); 7.63 (ddd, 1H, **H_7_**, *J_7-8_* = 8.3 Hz, *J_7-6_* = 7.0 Hz, *J_7-5_* = 1.5 Hz); 4.53 (d, 2H, C**H_2_**NH, *J_CH2-NH_* = 5.6 Hz); 4.07 (s, 3H, OC**H_3_**); 2.23 (q, 2H, C**H_2_**CH_3_, *J_CH2-CH3_* = 7.6 Hz); 1.06 (t, 3H, CH_2_C**H_3_**, *J_CH3-CH2_* = 7.6 Hz) ppm. Anal. Calcd for C_13_H_15_N_3_O_2_ · ^1^/_10_ H_2_O: C: 63.21%; H: 6.16%; N: 17.02%; Found: C: 63.44%; H: 6.08%; N: 16.65%. M.p.: 164 °C.

*N-(3-Methoxyquinoxalin-2-ylmethyl)-butyramide* (**3c**). Yield: 9%. IR (cm^−1^): 3309, 3065, 2951, 1641. ^1^H-NMR (DMSO-d_6_) *δ*: 8.30 (bs, 1H, N**H**); 7.93 (d, 1H, **H_8_**, *J_8-7_* = 7.9 Hz); 7.84 (d, 1H, **H_5_**, *J_5-6_* = 8.2 Hz); 7.70 (dd, 1H, **H_6_**, *J_6-5_* = 8.2 Hz, *J_6-7_* = 6.7 Hz); 7.61 (dd, 1H, **H_7_**, *J_7-8_* = 8.1 Hz, *J_7-6_* = 6.9 Hz); 4.52 (d, 2H, C**H_2_**NH, *J_CH2-NH_* = 5.4 Hz); 4.07 (s, 3H, OC**H_3_**); 2.18 (t, 2H, C**H_2_**CH_2_CH_3_, *J_CH2-CH2_* = 7.2 Hz); 1.56 (dd, 2H, CH_2_C**H_2_**CH_3_, *J_CH2-CH2_* = 7.2 Hz, *J_CH2-CH3_* = 7.2 Hz); 0.91 (t, 3H, C**H_3_**, *J_CH3-CH2_ =* 7.3 Hz) ppm. Anal. Calcd for C_14_H_17_N_3_O_2_·^1^/_8_ H_2_O: C: 64.31%; H: 6.60%; N: 16.08%; Found: C: 64.34%; H: 6.65%; N: 15.93%. M.p.: 259 °C.

*N-(3-Methoxyquinoxalin-2-ylmethyl)-isobutyramide* (**3d**).Yield: 28%. IR (cm^−1^): 3312, 3065, 2968, 2937, 1644. ^1^H-NMR (DMSO-d_6_) *δ*: 8.23 (t, 1H, N**H**, *J_NH-CH2_* = 5.6 Hz); 7.93 (dd, 1H, **H_8_**, *J_8-7_* = 8.2 Hz, *J_8-6_* = 1.0 Hz); 7.85 (dd, 1H, **H_5_**, *J_5-6_* = 8.2 Hz, *J_5-7_* = 0.9 Hz); 7.72 (ddd, 1H, **H_6_**, *J_6-5_* = 8.3 Hz, *J_6-7_* = 7.1 Hz, *J_6-8_* = 1.5 Hz); 7.62 (ddd, 1H, **H_7_**, *J_7-8_* = 8.4 Hz, *J_7-6_* = 7.1 Hz, *J_7-5_* = 1.5 Hz); 4.51 (d, 2H, C**H_2_**NH, *J_CH2-NH_* = 5.6 Hz); 4.07 (s, 3H, OC**H_3_**); 2.55 (q, 1H, C**H**(CH_3_)_2_, *J_CH-(CH3)2_* = 6.8 Hz); 1.08 (d, 6H, CH(C**H_3_**)**_2_**, *J_(CH3)2-CH_* = 6.8 Hz) ppm. Anal. Calcd for C_14_H_17_N_3_O_2_:C: 64.86%; H: 6.56%; N: 16.22%; Found: C: 64.55%; H: 6.79%; N: 15.91%. M.p.: 142–144 °C.

*N-(3-Methoxy-quinoxalin-2-ylmethyl)-benzamide* (**3e**). Yield: 38%. IR (cm^−1^): 3391, 3071, 3026, 2943, 2904, 1659. ^1^H-NMR (DMSO-d_6_) *δ*: 9.00 (t, 1H, N**H**, *J_NH-CH2_* = 5.4 Hz); 7.94-7.92 (m, 3H, **H_8_**+**H_2'_**+**H_6'_**); 7.86 (d, 1H, **H_5_**, *J_5-6_ =* 8.3 Hz); 7.71 (dd, 1H, **H_6_**, *J_6-5_ =* 8.1 Hz, *J_6-7_ =* 7.2 Hz); 7.63-7.47 (m, 4H, **H_7_**+**H_3'_**+**H_4'_**+**H_5'_**); 4.74 (d, 2H, C**H_2_**, *J_CH2-NH_* = 5.4 Hz); 4.10 (s, 3H, OC**H_3_**) ppm. Anal. Calcd for C_17_H_15_N_3_O_2_ · ^1^/_8_ H_2_O: C: 69.09%; H: 5.17%; N: 14.23%; Found: C: 69.13%; H: 5.14%; N: 13.99%. M.p.: 137 °C.

#### 3.1.5. General Procedure for Synthesis of 1-Alkyl-3-(3-methoxyquinoxalin-2-ylmethyl)ureas **3f–i**

Compound **2** (1 eq.), THF and the corresponding isocyanate (2 eq.) were placed in a hydrogenation tube. A spatula of Raney-Nickel was added and the reaction was carried out during 7 h at room temperature and 50 psi. Next, DCM was added and the mixture was decanted off with the aim of leaving behind the catalyst. Next, the mixture was quenched with water, dried over Na_2_SO_4_ and filtered. The solvent was removed under reduced pressure and the orange oil obtained was purified by flash chromatography using DCM/MeOH as mobile phase. The obtained solid was dissolved in water/diethyl ether with vigorous stirring and then quenched. The organic layer was dried over Na_2_SO_4_ and filtered. The solvent was removed under reduced pressure and the desired urea derivatives **3f**–**i**, were obtained.

*Ethyl-3-(3-methoxyquinoxalin-2-ylmethyl)urea* (**3f**). Yield: 3%. IR (cm^−1^): 3334, 3061, 2978, 2939, 1629. ^1^H-NMR (DMSO-d_6_) *δ*: 7.96 (dd, 1H, **H_8_**, *J_8-7_* = 8.2 Hz, *J_8-6_ =* 1.2 Hz); 7.85 (dd, 1H, **H_5_**, *J_5-6_* = 8.2 Hz, *J_5-7_ =* 1.2 Hz); 7.71 (ddd, 1H, **H_6_**, *J_6-5_* = 8.4 Hz, *J_6-7_* = 7.0 Hz, *J_6-8_ =* 1.4 Hz); 7.63 (ddd, 1H, **H_7_**, *J_7-8_* = 8.4 Hz, *J_7-6_* = 7.1 Hz, *J_7-5_ =* 1.4 Hz); 6.41 (t, 1H, ArCH_2_N**H**, *J_NH-CH2_ =* 5.0 Hz); 6.33 (t, 1H, N**H**CH_2_CH_3_, *J_NH-CH2_ =* 5.3 Hz); 4.50 (d, 2H, ArC**H_2_**NH, *J_CH2-NH_ =* 5.4 Hz); 4.07 (s, 3H, OC**H_3_**); 3.05 (dq, 2H, C**H_2_**CH_3_, *J_CH2-CH3_* = 7.2 Hz, *J_CH2-NH_* = 5.8 Hz); 1.02 (dt, 3H, C**H_3_**, *J_CH3-CH2_* = 7.2 Hz, *J_CH3-NH_* = 1.3 Hz) ppm. Anal. Calcd for C_13_H_16_N_4_O_2_: C: 60.00%; H: 6.15%; N: 21.54%; Found: C: 59.91%; H: 6.12%; N: 21.14%. M.p.: 160.5–160.8 °C.

*1-(3-Methoxyquinoxalin-2-ylmethyl)-3-propylurea* (**3g**). Yield: 9%. IR (cm^−1^): 3330, 3061, 2960, 1633. ^1^H-NMR (DMSO-d_6_) *δ*: 7.95 (d, 1H, **H_8_**, *J_8-7_* = 8.1 Hz); 7.85 (d, 1H, **H_5_**, *J_5-6_* = 8.2 Hz); 7.71 (dd, 1H, **H_6_**, *J_6-5_* = 8.0 Hz, *J_6-7_* = 7.3 Hz); 7.63 (dd, 1H, **H_7_**, *J_7-8_* = 8.0 Hz, *J_7-6_* = 7.1 Hz); 6.41 (t, 1H, ArCH_2_N**H**, *J_NH-CH2_ =* 5.5 Hz); 6.37 (t, 1H, N**H**CH_2_CH_2_CH_3_, *J_NH-CH2_ =* 6.0 Hz); 4.50 (d, 2H, ArC**H_2_**NH, *J_CH2-NH_ =* 5.2 Hz); 4.07 (s, 3H, OC**H_3_**); 2.98 (dd, 2H, NHC**H_2_**CH_2_CH_3_, *J_CH2-NH_ =* 6.4 Hz, *J_CH2-CH2_ =* 6.7 Hz); 1.40 (tq, 2H, NHCH_2_C**H_2_**CH_3_, *J_CH2-CH2_ =* 6.8 Hz, *J_CH2-CH3_ =* 7.1 Hz); 0.86 (t, 3H, C**H_3_**, *J_CH3-CH2_ =* 7.4 Hz) ppm. Anal. Calcd for C_14_H_18_N_4_O_2_: C: 61.31%; H: 6.57%; N: 20.44%; Found: C: 60.99%; H: 6.45%; N: 20.31%. M.p.: 115.3–116 °C.

*1-Isopropyl-3-(3-methoxyquinoxalin-2-ylmethyl)urea* (**3h**). Yield: 23%. IR (cm^−1^): 3363, 3283, 3100, 2971, 1620. ^1^H-NMR (DMSO-d_6_) *δ*: 7.95 (dd, 1H, **H_8_**, *J_8-7_* = 8.1 Hz, *J_8-6_ =* 1.1 Hz); 7.85 (dd, 1H, **H_5_**, *J_5-6_* = 8.2 Hz, *J_5-7_ =* 0.9 Hz); 7.71 (ddd, 1H, **H_6_**, *J_6-5_* = 8.2 Hz, *J_6-7_* = 7.7 Hz, *J_6-8_ =* 1.2 Hz); 7.63 (ddd, 1H, **H_7_**, *J_7-8_* = 8.0 Hz, *J_7-6_* = 7.4 Hz, *J_7-5_ =* 1.1 Hz); 6.34 (t, 1H, ArCH_2_N**H**, *J_NH-CH2_ =* 5.1 Hz); 6.26 (d, 1H, N**H**CH(CH_3_)_2_, *J_NH-CH_ =* 7.6 Hz); 4.49 (d, 2H, ArC**H_2_**NH, *J_CH2-NH_ =* 5.3 Hz); 4.07 (s, 3H, OC**H_3_**); 3.71–3.67 (m, 1H, C**H**(CH_3_)_2_); 1.06 (d, 6H, CH-(C**H_3_**)**_2_**, *J_(CH3)2-CH_* = 6.5 Hz) ppm. Anal. Calcd for C_14_H_18_N_4_O_2_: C: 61.31%; H: 6.57%; N: 20.44%; Found: C: 61.33%; H: 6.58%; N: 20.43%. M.p.: 171.2 °C.

*1-(3-Methoxyquinoxalin-2-ylmethyl)-3-phenyl-urea* (**3i**). Yield: 4%. IR (cm^−1^): 3310, 3177, 3023, 2952, 2907, 1640. ^1^H-NMR (DMSO-d_6_) *δ*: 9.01 (s, 1H, CON**H**-Ph); 7.99 (dd, 1H, **H_8_**, *J_8-7_ =* 8.2 Hz, *J_8-6_* = 1.2 Hz); 7.87 (dd, 1H, **H_5_**, *J_5-6_* = 8.3 Hz, *J_5-7_* = 1.1 Hz); 7.73 (ddd, 1H, **H_6_**, *J_6-5_* = 8.4 Hz, *J_6-7_* = 7.1 Hz, *J_6-8_ =* 1.5 Hz); 7.64 (ddd, 1H, **H_7_**, *J_7-8_* = 8.4 Hz, *J_7-6_* = 7.1 Hz, *J_7-5_ =* 1.5 Hz); 7.43 (dd, 2H, **H_2'_**+**H_6'_**, *J_2'-3'_* = *J_6'-5'_* = 8.6 Hz, *J_2'-4'_ = J_6'-4'_ =* 1.1 Hz); 7.24 (dd, 2H, **H_3'_**+**H_5'_**, *J_3'-2'_* = *J_5'-6'_* = 8.4Hz, *J_3'-4'_* = *J_5'-4'_* = 7.5 Hz); 6.91 (tt, 1H, **H_4'_**, *J_4'-3'_ = J_4'-5'_* = 7.5 Hz, *J_4'-2'_ = J_4'-6'_* = 1.1Hz); 6.83 (t, 1H, ArCH_2_N**H**, *J_NH-CH2_ =* 5.2 Hz); 4.61 (d, 2H, ArC**H_2_**NH, *J_CH2-NH_ =* 5.2 Hz); 4.10 (s, 3H, OC**H_3_**) ppm. Anal. Calcd for C_17_H_16_N_4_O_2_ · ^1^/_8_ H_2_O: C: 65.75%; H: 5.24%; N: 18.05%; Found: C: 65.76%; H: 5.32%; N: 18.12%. M.p.: 201–201.5 °C.

#### 3.1.6. Synthesis of 2,3-Dimethoxy-6-nitroquinoxaline (**4**)

2,3-Dichloro-6-nitroquinoxaline (5.12 g, 20.35 mmol) and THF (100.00 mL) were placed in a round bottomed flask. The mixture was cooled to 0 °C. NaOMe (9.6 mL, 41.00 mmol) prepared *in situ* by means of a reaction between Na (1.20 g) and MeOH (12.00 mL) was added dropwise to the other suspension. The color of the mixture changed from white-yellow to orange. The mixture was stirred during 30 min at 0 °C and during 1 h at room temperature. Then, DCM was added and the mixture was quenched with brine. The DCM solution was dried over Na_2_SO_4_ and filtered. The solvent was removed under reduced pressure in order to obtain compound **4** as a yellow solid. Yield: 52%. IR (cm^−1^): 3116, 3014, 2956. ^1^H-NMR (DMSO-d_6_) *δ*: 8.50 (d, 1H, **H_5_**, *J_5-7_* = 2.6 Hz); 8.30 (dd, 1H, **H_7_**, *J_7-8_* = 9.0 Hz, *J_7-5_* = 2.6 Hz); 7.93 (d, 1H, **H_8_**, *J_8-7_* = 9.0 Hz); 4.11 (s, 3H, OC**H_3_**_-C2_); 4.10 (s, 3H, OC**H_3_**_-C3_) ppm. Anal. Calcd for C_10_H_9_N_3_O_4_: C: 51.06%; H: 3.83%; N: 17.87%; Found: C: 50.99%; H: 3.79%; N: 17.40%.

#### 3.1.7. Synthesis of 6-Amino-2,3-dimethoxyquinoxaline (**5**)

Compound **4** (2.50 g, 10.64 mmol) was disolved in MeOH (150.00 mL) and the mixture was cooled to 0 °C. Then a tip of spatula of palladium on carbon and a great excess of hydrazine hydroxide were added. After stirring 30 min at 0 °C the mixture was stirred at room temperature for 15 h. The mixture was filtered to eliminate the catalyst and the solvent was removed under reduced pressure. Then, DCM was added and the mixture was quenched with brine. The DCM solution was dried over Na_2_SO_4_ and filtered. The solvent was removed under reduced pressure yielding to compound **5**. Yield: 96%. IR (cm^−1^): 3442, 3347, 3219, 2937. ^1^H-NMR (DMSO-d_6_) *δ*: 7.42 (d, 1H, **H_8_**, *J_8-7_ =* 8.6 Hz); 6.87 (dd, 1H, **H_7_**, *J_7-8_ =* 8.6 Hz, *J_7-5_ =* 2.4 Hz); 6.78 (d, 1H, **H_5_**, *J_5-7_ =* 2.5 Hz); 5.43 (s, 2H, N**H_2_**); 3.97 (s, 3H, OC**H_3_**_-C2_); 3.94 (s, 3H, OC**H_3_**_-C3_) ppm. Anal. Calcd for C_10_H_11_N_3_O_2_: C: 58.54%; H: 5.37%; N: 20.49%; Found: C: 58.08%; H: 5.37%; N: 20.41%.

#### 3.1.8. General Procedure for Synthesis of N-(2,3-Dimethoxyquinoxalin-6-yl)alkylamides **6a–e**

Compound **5** (1 eq.) was placed in suspension in THF. Next, the corresponding anhydride (3 eq.) and a catalytic amount of triethylamine were added dropwise. The reaction was carried out during 1 h 30 min at room temperature. THF was removed under reduced pressure. The obtained residue was treated with *n*-hexane and the obtained solid was then filtered. The solid was washed with water to yield the desired compounds **6a**–**e**. 

*N-(2*,*3-Dimethoxyquinoxalin-6-yl)acetamide* (**6a**). Yield: 13%. IR (cm^−1^): 3276, 3116, 2943, 1664. ^1^H-NMR (DMSO-d_6_) *δ*: 10.19 (s, 1H, N**H**); 8.21 (d, 1H, **H_5_**, *J_5-7_ =* 2.2 Hz); 7.67 (d, 1H, **H_8_**, *J_8-7_* = 8.8 Hz); 7.57 (dd, 1H, **H_7_**, *J_7-8_* = 8.9 Hz, *J_7-5_ =* 2.2 Hz); 4.03 (s, 3H, OC**H_3_**_-C2_); 4.01 (s, 3H, OC**H_3_**_-C3_); 2.10 (s, 3H, C**H_3_**) ppm. Anal. Calcd for C_12_H_13_N_3_O_3_ · ^1^/_2_ H_2_O: C: 56.25%; H: 5.47%; N: 16.41%; Found: C: 56.23%; H: 5.42%; N: 16.04%. M.p.: 290 °C.

*N-(2*,*3-Dimethoxyquinoxalin-6-yl)propionamide* (**6b**). Yield: 68%. IR (cm^−1^): 3284, 3175, 3105, 2987, 2943, 1663. ^1^H-NMR (DMSO-*d_6_*) *δ*: 10.11 (s, 1H, N**H**); 8.21 (s, 1H, **H_5_**); 7.67 (dd, 1H,**H_8_**, *J_8-7_* = 8.8 Hz, *J_8-5_* = 1.2 Hz); 7.60 (ddd, 1H, **H_7_**, *J_7-8_ =* 8.9 Hz, *J_7-5_ =* 2.2 Hz, *J_H7-NH_* = 1.5 Hz); 4.03 (d, 3H, OC**H_3_**_-C2_, *J_OCH3-OCH3_* = 1.4 Hz); 4.01 (d, 3H, OC**H_3_**_-C3_, *J_OCH3-OCH3_* = 1.4 Hz); 2.38 (dq, 2H, C**H_2_**, *J_CH2-CH3_ =* 7.5 Hz, *J_CH2-NH_ =* 1.3 Hz); 2.12 (dt, 3H,C**H_3_**, *J_CH3-CH2_ =* 7.5 Hz, *J_CH3-NH_ =* 1.4 Hz) ppm. Anal. Calcd for C_13_H_15_N_3_O_3_ · ^1^/_2_ H_2_O: C: 57.78%; H: 5.93%; N: 15.56%; Found: C: 58.15%; H: 5.92%; N: 15.61%. M.p.: 182–183 °C.

*N-(2*,*3-Dimethoxyquinoxalin-6-yl)butyramide* (**6c**). Yield: 26%. IR (cm^−1^): 3289, 2949, 1656. ^1^H-NMR (DMSO-d_6_) *δ*: 10.11 (s, 1H, N**H**); 8.21 (s, 1H, **H_5_**); 7.66 (dd, 1H, **H_7_**, *J_7-8_* = 8.4 Hz, *J_7-5_* = 2.9 Hz); 7.59 (d, 1H, **H_8_**, *J_8-7_ =* 8.7 Hz); 4.03 (d, 3H, OC**H_3_**_-C2_, *J_OCH3-OCH3_* = 2.6 Hz); 4.01 (d, 3H, OC**H_3_**_-C3_, *J_OCH3-OCH3_* = 2.6 Hz); 2.36–2.32 (m, 2H, COC**H_2_**CH_2_CH_3_); 1.66–1.62 (m, 2H, COCH_2_C**H_2_**CH_3_); 0.96–0.92 (m, 3H, C**H_3_**) ppm. Anal. Calcd for C_14_H_17_N_3_O_3_: C: 61.09%; H: 6.18%; N: 15.27%; Found: C: 60.78%; H: 6.32%; N: 15.59%. M.p.: 136–140 °C.

*N-(2*,*3-Dimethoxyquinoxalin-6-yl)isobutyramide* (**6d**). Yield: 72%. IR (cm^−1^): 3253, 3026, 2969, 1653. ^1^H-NMR (DMSO-*d_6_*) *δ*: 10.07 (s, 1H, N**H**); 8.21 (d, 1H, **H_5_**, *J_5-7_ =* 2.2 Hz); 7.67 (d, 1H, **H_8_**, *J_8-7_* = 8.9 Hz); 7.62 (dd, 1H,**H_7_**, *J_7-8_ =* 8.9 Hz, *J_7-5_* = 2.3 Hz); 4.03 (d, 3H, OC**H_3_**_-C2_, *J_OCH3-OCH3_* = 1.2 Hz); 4.01 (d, 3H, OC**H_3_**_-C3_, *J_OCH3-OCH3_* = 1.3 Hz); 2.64 (dq, 1H, C**H**(CH_3_)_2_, *J_CH-(CH3)2_ =* 6.7 Hz); 1.14 (d, 6H, CH(C**H_3_**)**_2_**, *J_(CH3)2-CH_* = 6.8 Hz) ppm. Anal. Calcd for C_14_H_17_N_3_O_3_ · ^1^/_8_ H_2_O: C: 60.60%; H: 6.22%; N: 15.15%; Found: C: 60.60%; H: 6.07%; N: 15.12%. M.p.: 201–203 °C.

*N-(2*,*3-Dimethoxyquinoxalin-6-yl)benzamide* (**6e**). Yield: 42%. IR (cm^−1^): 3285, 3061, 2984, 2939, 1651. ^1^H-NMR (DMSO-d_6_) *δ*: 10.49 (s, 1H, N**H**); 8.37 (d, 1H, **H_5_**, *J_5-7_ =* 2.2 Hz); 8.00–7.98 (m, 2H, **H_2'_**+**H_6'_**); 7.87 (dd, 1H, **H_7_**, *J_7-8_ =* 8.9 Hz, *J_7-5_* = 2.4 Hz); 7.73 (d, 1H, **H_8_**, *J_8-7_ =* 8.8 Hz); 7.65–7.53 (m, 3H, **H_3'_**+**H_4'_**+**H_5'_**); 4.05 (s, 3H, OC**H_3_**_-C2_, *J_OCH3-OCH3_* = 0.5 Hz); 4.03 (s, 3H, OC**H_3_**_-C3_, *J_OCH3-OCH3_* = 0.5 Hz) ppm. Anal. Calcd for C_17_H_15_N_3_O_3_ · ^1^/_8_ H_2_O: C: 65.54%; H: 4.90%; N: 13.49%; Found: C: 65.52%; H: 4.99%; N: 13.40%. M.p.: 165–170 °C.

#### 3.1.9. General Procedure for Synthesis of 1-(2,3-Dimethoxyquinoxaline-6-yl)-3-alkylureas **6f–i**

Compound **5** (1 eq.) was dissolved in dry DCM (15 mL) and placed under N_2_ atmosphere. The mixture was cooled to 0 °C and the corresponding isocyanate (2 eq.) was added. The reaction was carried out during 30 min at 0 °C and 3 h at room temperature. Next, the obtained precipitate was filtered to achieve the corresponding compounds **6f**–**i**.

*1-(2*,*3-Dimethoxyquinoxalin-6-yl)-3-ethylurea* (**6f**). Yield: 24%. IR (cm^−1^): 3303, 3103, 2981, 1633. ^1^H-NMR (DMSO-d_6_) *δ*: 8.69 (s, 1H, CON**H**Ar); 7.97 (d, 1H, **H_5_**, *J_5-7_ =* 2.2 Hz); 7.60 (d, 1H, **H_8_**, *J_8-7_* = 8.8 Hz); 7.41 (dd, 1H*,* **H_7_**, *J_7-8_ =* 8.8 Hz, *J_7-5_* = 2.5 Hz); 6.20 (t, 1H, CON**H**CH_2_, *J_NH-CH2_* = 5.4 Hz); 4.02 (d, 3H, OC**H_3_**_-C2_, *J_OCH3-OCH3_* = 0.9 Hz); 3.99 (s, 3H, OC**H_3_**_-C3_, *J_OCH3-OCH3_* = 0.9 Hz); 3.14 (dq, 2H, C**H_2_**, *J_CH2-NH_* = 5.9 Hz, *J_CH2-CH3_ =* 7.2 Hz); 1.08 (t, 3H, C**H_3_**, *J_CH3-CH2_ =* 7.3 Hz) ppm. Anal. Calcd for C_13_H_16_N_4_O_3_ · ^1^/_4_ H_2_OC: 55.61%; H: 5.88%; N: 19.96%; Found: C: 55.57%; H: 5.66%; N: 19.94%. M.p.: **>**300 °C.

*1-(2*,*3-Dimethoxyquinoxalin-6-yl)-3-propylurea* (**6g**). Yield: 29%. IR (cm^−1^): 3289, 2949, 1656. ^1^H-NMR (DMSO-d_6_) *δ*: 8.67 (s, 1H, CON**H**Ar); 7.96 (dd, 1H, **H_5_**, *J_5-7_ =* 2.2 Hz, *J_5-8_ =* 0.6 Hz); 7.60 (dd, 1H, **H_8_**, *J_8-7_* = 8.8 Hz, *J_8-5_ =* 0.6 Hz); 7.40 (ddd, 1H*,* **H_7_**, *J_7-8_ =* 8.9 Hz, *J_7-5_* = 2.4 Hz, *J_H7-NH_* = 0.8 Hz); 6.24 (t, 1H, CON**H**CH_2_, *J_NH-CH2_* = 5.8 Hz); 4.02 (s, 3H, OC**H_3_**_-C2_, *J_OCH3-OCH3_* = 0.9 Hz); 3.99 (s, 3H, OC**H_3_**_-C3_, *J_OCH3-OCH3_* = 0.9 Hz); 3.08 (dd, 2H, NHC**H_2_**CH_2_CH_3_, *J_CH2-CH2_* = 6.4 Hz, *J_CH2-NH_* = 6.3 Hz); 1.47 (tq, 2H, NHCH_2_C**H_2_**CH_3_, *J_CH2-CH3_ =* 7.3 Hz, *J_CH2-CH2_ =* 7.2 Hz); 0.89 (t, 3H, C**H_3_**, *J_CH3-CH2_ =* 7.4 Hz) ppm. Anal. Calcd for C_14_H_18_N_4_O_3_: C: 57.93%; H: 6.21%; N: 19.31%; Found: C: 57.78%; H: 6.11%; N: 19.31%. M.p.: 122–123 °C.

*1-(2*,*3-Dimethoxyquinoxalin-6-yl)-3-isopropylurea* (**6h**). Yield: 10%. IR (cm^−1^): 3334, 3297, 3097, 2962, 1637. ^1^H-NMR (DMSO-d_6_) *δ*: 8.55 (s, 1H, CON**H**Ar); 7.95 (dd, 1H, **H_5_**, *J_5-7_ =* 2.3 Hz, *J_5-8_ =* 1.2 Hz); 7.60 (dd, 1H, **H_8_**, *J_8-7_* = 8.9 Hz, *J_8-5_* = 1.0 Hz); 7.39 (ddd, 1H,**H_7_**, *J_7-8_ =* 8.9 Hz, *J_7-5_* = 2.4 Hz, *J_H7-NH_* = 1.1 Hz); 6.10 (d, 1H, CON**H**CH(CH_3_)_2_, *J_NH-CH_* = 7.7 Hz); 4.02 (s, 3H, OC**H_3_**_-C2_, *J_OCH3-OCH3_* = 1.3 Hz); 3.99 (s, 3H, OC**H_3_**_-C3_, *J_OCH3-OCH3_* = 1.3 Hz); 3.84–3.75 (m, 1H, C**H**); 1.12 (dd, 6H, CH(C**H_3_**)**_2_**, *J_(CH3)2-CH_ =* 6.5 Hz, *J_(CH3)2-NH_ =* 1.2 Hz) ppm. Anal. Calcd for C_14_H_18_N_4_O_3_ · ^1^/_4_ H_2_O: C: 57.05%; H: 6.28%; N: 19.02%; Found: C: 57.12%; H: 6.24%; N: 18.88%. M.p.: 250 °C.

*1-(2*,*3-Dimethoxyquinoxalin-6-yl)-3-phenylurea* (**6i**). Yield: 55%. IR (cm^−1^): 3275; 3068, 3030, 2997, 2939; 1639. ^1^H-NMR (DMSO-d_6_) *δ*: 8.94 (s, 1H, N**H**); 8.77 (s, 1H, N**H**); 8.02 (d, 1H, **H_5_**, *J_5-7_ =* 2.1 Hz); 7.66 (d, 1H, **H_8_**, *J_8-7_ =* 8.8 Hz); 7.54–7.43 (m, 3H, **H_7_**+**H_2'_**+**H_6'_**); 7.30 (dd, 2H, **H_3'_**+**H_5'_**, *J_3'-2'_* = *J_5'-6'_* = 8.1Hz, *J_3'-4'_* = *J_5'-4'_* = 7.7 Hz); 6.99 (t, 1H, **H_4’_**, *J_4'-3'_ = J_4'-5'_* = 7.4 Hz); 4.03 (s, 3H, OC**H_3_**_-C2_); 4.00 (s, 3H, OC**H_3_**_-C3_) ppm. Anal. Calcd for C_17_H_16_N_4_O_3_: C: 62.96%; H: 4.94%; N: 17.28%; Found: C: 63.30%; H: 5.23%; N: 17.56%. M.p.: 260 °C.

#### 3.1.10. Synthesis of 3-Amino-7-methoxy-1,4-di-N-oxidequinoxaline-2-carbonitrile (**7**)

5-Methoxybenzofuroxan (2.50 g, 15.06 mmol), malononitrile (1.80 mL, 18.00 mmol) and *N,N-*DMF (5.00 mL) were placed in a flask. Next, the mixture was cooled to 0 °C and triethylamine (1.50 mL, 10.77 mmol) was added dropwise. The reaction was carried out during 6 h at room temperature. Diethyl ether and methanol were added and the mixture was filtered. The obtained compound was used without further purification. This compound has already been described by Monge *et al*. [24].

#### 3.1.11. Synthesis of 7-Methoxy-1,4-di-N-oxidequinoxaline-2-carbonitrile (**8**)

Compound **7** (1.60 g, 6.88 mmol) and *N,N-*DMF (30.00 mL) were placed in a flask under a N_2_ atmosphere. Next, the mixture was heated to 65 °C and *tert-*butyl nitrite (2.80 mL, 23.57 mmol) was added dropwise. Effervescence could be observed. After stirring 10 min at 65 °C, another portion of *tert-*butyl nitrite (2.10 mL, 17.68 mmol) was added and the mixture was stirred for 1 h 30 min. The solvent was removed under reduced pressure. Finally, the solid was purified by traditional column chromatography using DCM as mobile phase to yield compound **8**. Yield: 31%. IR (cm^−1^): 3090, 2975, 2238, 1367. ^1^H-NMR (DMSO-d_6_) *δ*: 9.19 (s, 1H, **H_3_**); 8.38 (d, 1H, **H_5_**, *J_5-6_* = 9.4 Hz); 7.74 (d, 1H, **H_8_**, *J_8-6_* = 2.7 Hz); 7.69 (dd, 1H, **H_6_**, *J_6-5_* = 9.4 Hz, *J_6-8_* = 2.8 Hz); 4.02 (s, 3H, OC**H_3_**) ppm. Anal. Calcd for C_10_H_7_N_3_O_3_ · ^1^/_8_ H_2_O: C: 54.73%; H: 3.31%; N: 19.16%; Found: C: 54.86%; H: 3.30%; N: 18.85%.

#### 3.1.12. Synthesis of 7-Methoxyquinoxaline-2-carbonitrile (**9**)

Compound **8** (0.91 g, 0.97 mmol) and AcOEt/MeOH (1:1, 40.00 mL) were placed in a flask and heated to 65 °C. Next, a solution of sodium dithionite in water was prepared *in situ* (1.01 g of Na_2_O_4_S_2_in 20.00 mL of water) and added over the mixture. The reaction was carried out during 10 min at 65 °C. Next, the mixture was filtered with the aim of removing the sodium dithionite, and the solvent was removed under reduced pressure. Finally, the crude residue was purified by flash column chromatography to yield compound **9**. Yield: 39%. IR (cm^−1^): 3090, 3039, 2975, 2226. ^1^H-NMR (DMSO-d_6_) *δ*: 9.22 (s, 1H, **H_3_**); 8.13 (d, 1H, **H_5_**, *J_5-6_* = 9.3 Hz); 7.73 (dd, 1H, **H_6_**, *J_6-5_* = 9.3 Hz, *J_6-8_* = 2.8 Hz); 7.58 (d, 1H, **H_8_**, *J_8-6_* = 2.8 Hz); 4.00 (s, 3H, OC**H_3_**) ppm. Anal. Calcd for C_10_H_7_N_3_O · ^1^/_8_ H_2_O: C: 64.09%; H: 3.87%; N: 22.43%; Found: C: 64.36%; H: 3.73%; N: 22.29%.

#### 3.1.13. General Procedure for Synthesis of N-[(7-Methoxyquinoxalin-2-yl)methyl]alkylamides **10a–e**

Compound **9** (1 eq.), THF (20 mL) and the corresponding anhydride (2 eq.) were placed in a hydrogenation flask. A spatula of Raney-Nickel was added and the mixture was hydrogenated during 7 h at 50 °C and 50 psi. Next, DCM was added and the mixture was decanted off with the aim of leaving off the catalyst. Next, the mixture was quenched with water, dried over Na_2_SO_4_ and filtered. The solvent was removed under reduced pressure and the orange oil obtained was purified through flash chromatography in order to obtain the amide derivatives **10a**–**e**. 

*N-[(7-Methoxyquinoxalin-2-yl)methyl]acetamide* (**10a**). Yield: 6%. IR (cm^−1^): 3289, 3068, 2965, 2920, 1648. ^1^H-NMR (DMSO-d_6_) *δ*: 8.71 (s, 1H, **H_3_**); 8.62 (t, 1H, N**H**, *J_NH-CH2_* = 5.3 Hz); 7.97 (d, 1H,**H_5_**, *J_5-6_* = 9.1 Hz); 7.62 (dd, 1H, **H_6_**, *J_6-5_ =* 9.1 Hz, *J_6-8_ =* 2.8 Hz); 7.40 (d, 1H,**H_8_**, *J_8-6_* = 2.6 Hz); 4.56 (d, 2H, C**H_2_**NH, *J_CH2-NH_* = 5.8 Hz); 3.95 (s, 3H, OC**H_3_**); 1.93 (s, 3H, C**H_3_**) ppm. Anal. Calcd for C_12_H_13_N_3_O_2_ · ^1^/_8_ H_2_O: C: 61.74%; H: 5.68%; N: 18.01%; Found: C: 61.99%; H: 5.69%; N: 17.67%. M.p.: 123.9–126.9 °C.

*N-[(7-Methoxyquinoxalin-2-yl)methyl]propionamide* (**10b**). Yield: 24%. IR (cm^−1^): 3280, 3068, 2971, 2913, 1643. ^1^H-NMR (DMSO-d_6_) *δ*: 8.70 (s, 1H, **H_3_**); 8.54 (t, 1H, N**H**, *J_CH2-NH_ =* 5.6 Hz); 7.97 (d, 1H, **H_5_**, *J_5-6_ =* 9.1 Hz); 7.46 (dd, 1H, **H_6_**, *J_6-5_ =* 9.1 Hz, *J_6-8_* = 2.8 Hz); 7.40 (d, 1H, **H_8_**, *J_8-6_ =* 2.8 Hz); 4.56 (d, 2H, C**H_2_**NH, *J_CH2-NH_ =* 5.8 Hz); 3.95 (s, 1H, OC**H_3_**); 2.21 (q, 2H, C**H_2_**CH_3_, *J_CH2-CH3_ =* 7.6 Hz); 1.04 (t, 3H, C**H_3_**, *J_CH3-CH2_ =* 7.6 Hz) ppm. Anal. Calcd for C_13_H_15_N_3_O_2_ · ^1^/_4_ H_2_O: C: 62.53%; H: 6.21%; N: 16.83%; Found: C: 62.92%; H: 6.23%; N: 16.85%. M.p.: 143–145 °C.

*N-[(7-Methoxyquinoxalin-2-yl)methyl]butyramide* (**10c**). Yield: 24%. IR (cm^−1^): 3282, 3081, 2965, 1643. ^1^H-NMR (DMSO-d_6_) *δ*: 8.69 (s, 1H, **H_3_**); 8.58 (s, 1H, N**H**); 7.96 (d, 1H, **H_5_**, *J_5-6_* = 8.7 Hz); 7.45 (d, 1H, **H_6_**, *J_6-5_* = 8.4 Hz); 7.38 (s, 1H, **H_8_**); 4.56 (d, 2H, C**H_2_**NH, *J_CH2-NH_* = 4.8 Hz); 3.94 (s, 3H, OC**H_3_**); 2.17 (t, 2H C**H_2_**CH_2_CH_3_, *J_CH2-CH2_* = 6.5 Hz*)*; 1.60–1.50 (m, 2H, CH_2_C**H_2_**CH_3_); 0.88 (t, 3H, C**H_3_**, *J_CH3-CH2_* = 6.6 Hz) ppm. Anal. Calcd for C_14_H_17_N_3_O_2_ · ^1^/_2_ H_2_O: C: 62.69%; H: 6.72%; N: 15.67%; Found: C: 62.49%; H: 6.55%; N: 15.53%. M.p.: 128–129 °C.

*N-[(7-Methoxyquinoxalin-2-yl)methyl]isobutyramide* (**10d**). Yield: 62%. IR (cm^−1^): 3280, 3074, 2971, 2926, 1648. ^1^H-NMR (DMSO-d_6_) *δ*: 8.67 (s, 1H, **H_3_**); 8.53 (t, 1H, N**H**, *J_NH-CH2_ =* 5.3 Hz); 7.97 (d, 1H, **H_5_**, *J_5-6_ =* 9.2 Hz); 7.46 (dd, 1H, **H_6_**, *J_6-5_ =* 9.2 Hz, *J_6-8_* = 2.8 Hz); 7.39 (d, 1H, **H_8_**, *J_8-6_* = 2.7 Hz); 4.55 (d, 2H, C**H_2_**NH, *J_CH2-NH_ =* 5.8 Hz); 3.95 (s, 3H, OC**H_3_**); 2.52–2.48 (m, 1H, C**H**); 1.07 (d, 6H, CH**(CH_3_)_2_**, *J_(CH3)2-CH_* = 6.9 Hz) ppm. Anal. Calcd for C_14_H_17_N_3_O_2_: C: 64.86%; H: 6.56%; N: 16.22%; Found: C: 64.76%; H: 6.67%; N: 16.27%. M.p.: 133–133.5 °C.

*N-[(7-Methoxyquinoxalin-2-yl)methyl]benzamide* (**10e**). Yield: 29%. IR (cm^−1^): 3299, 3061, 3010, 2965, 2920, 1640. ^1^H-NMR (DMSO-d_6_) *δ*: 9.28 (t, 1H, N**H**, *J_NH-CH2_* = 5.4 Hz); 8.79 (s, 1H, **H_3_**); 7.98 (d, 1H, **H_5_**, *J_5-6_* = 9.1 Hz); 7.94 (dd, 2H, **H_2'_**+**H_6'_**, *J_2'-3'_* = *J_6'-5'_* = 7.1Hz, *J_2'-4'_* = *J_6'-4'_* = 1.2 Hz); 7.59-7.44 (m, 4H, **H_6_**+**H_3'_**+**H_4'_**+**H_5'_**); 7.41 (d, 1H,**H_8_**, *J_8-6_* = 2.7 Hz); 4.79 (d, 2H, C**H_2_**NH, *J_CH2-NH_* = 5.7 Hz); 3.94 (s, 3H, OC**H_3_**) ppm. Anal. Calcd for C_17_H_15_N_3_O_2_ · ^1^/_4_ H_2_O: C: 68.57%; H: 5.21%; N: 14.12%; Found: C: 68.66%; H: 5.35%; N: 13.95%. M.p.: 142–143.9 °C.

### 3.2. Pharmacology

#### 3.2.1. Reagents and Chemicals

2-[^125^I]Iodomelatonin (2200 Ci/mmol) was purchased from NEN (Boston, MA, USA). Other drugs and chemicals were purchased from Sigma-Aldrich (Saint Quentin, France).

#### 3.2.2. Cell Culture

HEK (provided by A.D. Strosberg, Paris, France) and CHO cell lines stably expressing the human melatonin MT_1_ or MT_2_ receptors were grown in DMEM medium supplemented with 10% fetal calf serum, 2 mM glutamine, 100 IU/mL penicillin and 100 µg/mL streptomycin. Grown at confluence at 37 °C (95% O_2_/5% CO_2_), they were harvested in PBS containing EDTA 2 mM and centrifuged at 1,000 g for 5 min (4 °C). The resulting pellet was suspended in Tris 5 mM (pH 7.5), containing EDTA 2 mM and homogenized using a Kinematica polytron. The homogenate was then centrifuged (95,000 g, 30 min, 4 °C) and the resulting pellet suspended in 75 mM Tris (pH 7.5), 12.5 mM MgCl_2_ and 2 mM EDTA. Aliquots of membrane preparations were stored at −80 °C until use.

#### 3.2.3. Binding Assays

2-[^125^I]Iodomelatonin binding assay conditions are essentially as previously described [50]. Briefly, binding is initiated by the addition of membrane preparations from stable transfected HEK or CHO cells diluted in binding buffer (50 mM Tris-HCl buffer, pH 7.4, containing 5 mM MgCl_2_) to 2-[^125^I]Iodomelatonin (25 or 200 pM for MT_1_ and MT_2_ receptors, respectively, expressed in HEK cells or 20 pM for MT_1_ and MT_2_ receptors expressed in CHO cells) and the tested drug. Non-specific binding was defined in the presence of 1 µM melatonin. After 120 min of incubation at 37 °C, the reaction was stopped by rapid filtration through GF/B filters presoaked in 0.5% (v/v) polyethylenimine. Filters were washed three times with 1 mL of ice-cold 50 mM Tris-HCl buffer, pH 7.4. Data from dose-response curves (seven concentrations in duplicate) were analyzed using the program PRISM (Graph Pad Software Inc., San Diego, CA, USA) to yield IC_50_ (inhibitory concentration 50). Results were expressed as Ki using the Cheng and Prusoff equation: Ki = IC_50_/[1 + (L/K_D_)], where [L] is the concentration of radioligand used in the assay and K_D_, the dissociation constant of the radioligand characterizing the membrane preparation.

The equilibrium binding constant (K_D_) for the 2-iodomelatonin at hMT_1_ and hMT_2_ receptors expressed in CHO and HEK cell membranes were 21 ± 3 pM and 107 ± 11 pM (*n = 4*), for CHO-hMT_1_ and CHO-hMT_2_ membranes, respectively and 10 ± 1 pM and 83 ± 19 pM (*n = 4*) for HEK-hMT_1_ and HEK-hMT_2_, respectively.

[^35^S]GTPγS binding assay was performed according to published methodology [50]. Briefly, membranes from transfected CHO cells expressing MT_1_ and MT_2_ receptor subtypes and compounds were diluted in binding buffer (20 mM HEPES, pH 7.4, 100 mM NaCl, 3 μM GDP, 3 mM MgCl_2_, and 20 μg/mL saponin). Incubation was started by the addition of 0.2 nM [^35^S]GTPγS to membranes (20 μg/mL) and drugs, and further followed for 1h at room temperature. 

Usual levels of [^35^S]GTPγS binding (expressed in dpm) were for CHO-MT_2 _membranes: 2,000 for basal activity, 8,000 in the presence of melatonin 1 μM and 180 in the presence of GTPγS 10 μM which defined the non-specific binding. Data from the dose-response curves (seven concentrations in duplicate) were analyzed by using the program PRISM (Graph Pad Software Inc., San Diego, CA, USA) to yield EC_50_ (effective concentration 50%) and E_max_ (maximal effect) for agonists.

## 4. Conclusions

In conclusion, we were not able to assert whether the quinoxaline moiety can replace the melatonin indole nucleus as a useful bioisostere in the search of new melatoninergic ligands. Series III showed the best binding affinities to MT_1_/MT_2_ receptors due to the fact that the distance between the methoxy group and the nitrogen atom of the amide/urea group is comparable to the distance in the MLT molecule, a six-atom distance. Compound **10c** showed the best binding affinity to both receptors. We found a large selectivity on the MT_2_ receptor subtype by switching the methoxy group to position 2 and establishing a six-atom distance between this group and *N*-acylamido chain (Series II), displaying a very selective MT_2_ ligand with a MT_2_/MT_1_ ratio of 250 and the higher binding affinity to MT_2_ (0.08 μM) of this study. This may provide a good preliminary starting point taking into consideration the new structural characteristics were set up as essential requirements for obtaining new quinoxalines as potent MT_1_ and MT_2_ receptor ligands. 
